# Expulsion rates 12 months after early versus interval postpartum intrauterine device placement: a randomized trial

**DOI:** 10.1016/j.xagr.2025.100547

**Published:** 2025-07-20

**Authors:** Sarah Averbach, Florin Vaida, Erica Hinz, Gennifer Kully, Arnab K. Dey, Monica A. Lutgendorf, Sadia Haider, Lisa G. Hofler

**Affiliations:** a Department of Obstetrics, Gynecology and Reproductive Sciences, Division of Complex Family Planning, University of California, San Diego, CA (Averbach, and Kully); b Center on Gender Equity and Health, University of California, San Diego, CA (Averbach, Kully, and Dey); c Division of Biostatistics, School of Public Health, University of California, San Diego, CA (Vaida); d Department of Obstetrics and Gynecology, Division of Complex Family Planning, University of Illinois at Chicago, Chicago, IL (Hinz); e Department of Gynecologic Surgery & Obstetrics, Uniformed Services University of the Health Sciences, Bethesda, MD (Lutgendorf); f Department of Obstetrics and Gynecology, Division of Complex Family Planning, University of Chicago, Chicago, IL (Haider); g Department of Obstetrics and Gynecology, Division of Complex Family Planning, University of New Mexico, Albuquerque, NM (Hofler)

**Keywords:** early, expulsion, intrauterine device, postpartum

## Abstract

**Objective:**

The early postpartum period, 2 to 4 weeks postpartum, is an optimal time for intrauterine device (IUD) initiation; placement can be co-located with early postpartum or infant visits. We aimed to compare expulsion rates at 12 months postpartum for IUDs placed early compared to the standard interval 6-week visit.

**Study Design:**

This is a randomized controlled trial conducted at four U.S. medical centers. Participants were randomly assigned to early (14–28 days) or interval (42–56 days) postpartum IUD placement after vaginal or cesarean birth. We used transvaginal ultrasound to confirm IUD presence and position at 6 months. Chart review and telephone surveys were used to verify IUD presence and position at 12 months.

**Results:**

Between March 2018 and June 2021, 203 participants were assigned to early and 201 to interval IUD placement; 238 (58.9%) contributed outcome data by phone survey (and electronic medical record review) at 12-months postpartum. Among participants who received an IUD and provided 12-month outcome data, complete expulsion rates were 4 in 124 (3.2%; 95% confidence interval [CI], 0.90 to 8.2) and 0 in 114 (0%; 95% CI, 0 to 3.2) in the early and interval groups; a between-group difference of 3.2 percentage points (95% CI, −0.01 to 8.0, *P*=.054). Partial expulsion counts and rates were 16 (12.9%; 95% CI, 7.6 to 20.1) and 13 (11.4%; 95% CI, 6.2 to 18.7) in the early and interval groups; a difference of 1.5 percentage points (95% CI, −7.2 to 10.2, *P*=.75). Among all 404 participants, IUD utilization rates at 12-month follow-up were 113 (55.7%; 95% CI, 48.5 to 62.6 among participants in the early group) compared to 95 (47.3%; 95% CI, 40.2 to 54.1, *P*=.10 among participants in the interval group). Participants were more satisfied with early compared to interval placement, 107 (86.3%; 95% CI 79.0 to 91.8) vs 87 (76.3%, 95% CI 67.4 to 83.8 95%) *P*=.048**.**

**Conclusion:**

Complete expulsion rates at 12 months are low (<5%) when IUDs are placed in the early and interval postpartum period. Satisfaction is higher with early postpartum IUD placement.

**AJOG Global Reports at a Glance**

**Why was this study conducted?**

To understand the rates of complete expulsion after intrauterine devices (IUDs) are placed early in the postpartum period, 2 to 4 weeks after birth.

**Key findings**

In this randomized clinical trial that included 404 postpartum people, the rate of complete expulsion at 12 months postpartum after early postpartum IUD was similar to the expulsion rate after interval placement 6 to 8 weeks after birth (3%vs 0%).

**What does this add to what is known?**

IUD expulsions can be diagnosed over time following placement. When devices are placed early, at 2 to 4 weeks, compared with 6 to 8 weeks postpartum, complete expulsion rates remain low for the first 12 months.

## Introduction

The early postpartum (EPP) period, 2 to 4 weeks postpartum, could be a convenient time to receive contraception, including intrauterine devices (IUDs). People are not yet at risk for pregnancy and the visit can be co-located with other visits, such as infant visits.^1^ The American College of Obstetricians and Gynecologists (ACOG) recommends all postpartum people have contact with an obstetrical provider within the first several weeks of delivery, so patients will increasingly be visiting with clinicians in the EPP period.^2^

Studies have shown that EPP IUD placement is feasible and acceptable.^3^, ^4^, ^5^ A meta-analysis found no complete expulsions and a 3.7% partial expulsion risk when IUDs are placed in the EPP period, but this analysis was limited by the small number of studies which included only 136 participants.^6^ We evaluated the rates of complete IUD expulsion after early postpartum placement, 2 to 4 weeks after birth, compared with interval postpartum placement 6 to 8 weeks after birth, in a randomized controlled trial. We found that the rate of complete expulsion at 6 months postpartum after early postpartum IUD placement (2–4 weeks postpartum) was noninferior to the rate of expulsion after interval postpartum placement (6–8 weeks postpartum) (2% vs 0%) based on a noninferiority margin of 6%.^7^ However, data suggest that expulsions after postpartum IUD placement are sometimes diagnosed beyond the first few months postpartum.^8^ Therefore, in this planned secondary outcome analysis we aimed to compare the 12-month expulsion rate of EPP IUDs inserted to the standard interval placement timing.

## Materials and methods

We conducted a randomized noninferiority trial conducted between March 2018 and June 2021 at University of California, San Diego, University of Illinois at Chicago, Naval Medical Center of San Diego, and University of New Mexico, and described the methods elsewhere in detail.^7^

Briefly, we assessed patients for eligibility who indicated that they desired an IUD for postpartum contraception. We recruited participants who were 18 years and older, spoke English or Spanish, and had a vaginal or cesarean delivery within 10 days. We excluded those who had any medical contraindication to an IUD per the US CDC Medical Eligibility Criteria.^9^

Participants provided written informed consent and then completed a brief self-administered baseline survey on a tablet including self-reported demographics (age, ethnicity, race, education, parity, employment, and type of delivery). After enrollment, we randomized participants 1:1 to early postpartum IUD placement (14–28 days after delivery) or interval placement (42–56 days after delivery). Participants chose what type of IUD they wanted (hormonal or copper).

Participants in the EPP group were scheduled for an IUD placement between 14 and 28 days postpartum. IUDs were placed by the participant’s usual prenatal provider or any clinician that the participant would normally be referred to for IUD placement. Use of ultrasound guidance for placement or for confirmation of position after insertion was at the discretion of the clinician.

All participants were scheduled for a postpartum visit at 6 weeks postpartum. Participants randomized to interval placement were scheduled for IUD placement between 42 and 56 days postpartum, typically at the time of the 6-week postpartum visit.

At the 6-month follow up study visit, a study clinician blinded to study group conducted a bimanual pelvic exam, speculum exam, and transvaginal ultrasonography. Participants completed a 6-month study survey assessing any expulsions, IUD replacements, perforations or infections, pregnancies, and satisfaction with the timing of IUD placement. We confirmed any reported outcomes with chart review when possible.

The primary outcome was complete expulsion (no IUD seen within the uterus on transvaginal ultrasound and either a clinical history consistent with certain expulsion and/or an abdominal and pelvic x-ray confirming absence of the IUD). Other outcomes included partial expulsion (IUD protruding from the external cervical os on speculum exam or any part of the IUD seen below the internal cervical os on transvaginal ultrasound) and malposition (low lying or rotated IUD).

At 12 months postpartum we called every participant and conducted a brief telephone survey assessing for any further expulsions, IUD replacements, perforations or infections, pregnancies that occurred between 6 and 12 months postpartum, and continued satisfaction with the IUD and timing of IUD placement. We also reviewed each participant’s electronic medical record to assess for any IUD-related visits. We attempted to contact participants between 365 and 486 days (12–16 months) postpartum by phone, calling up to 3 times. We considered participants lost to follow-up if they did not respond after 3 contact attempts or if 487 days passed without contact.

The sample size was based on the primary outcome of complete expulsion at 6-months postpartum (7). We estimated that a minimum sample size of 336 (168 in each group) would be required. We presented analyses of outcome data from the 6-month study visits elsewhere.^7^

The primary outcome for this planned analysis, complete expulsion by 12-months postpartum, was evaluated per modified intent-to-treat analysis excluding those not at risk for expulsion because they did not have an IUD placed. Secondary analyses (utilization, pregnancy) were planned using a true intent-to-treat evaluating all randomized participants and a per-protocol approach evaluating expulsions among participants who had an IUD placed during the time frame they were randomized to: 14 to 28 days (early) or 42 to 56 days after delivery (interval).

We compared groups using an exact z-pooled test with exact CIs. Categorical variables were compared using chi-square test or Fisher’s exact test when a cell contained fewer than five observations. Study data were collected and managed using REDCap electronic data capture tools.^10^ Data analyses were completed using R (version 4.2.1).

This study was approved by the Institutional Review Boards at University of California, San Diego, University of Illinois at Chicago and University of New Mexico. The Naval Medical Center relied on University of California San Diego’s approval.

## Results

Briefly, between March 2018 and July 2021, we randomized 203 (50.2%) participants to the early group and 201 (49.8%) to the interval group; 325 (80.4%) received an IUD by 12 months postpartum (163 early, 162 interval). Of those participants, 238 (58.9%) contributed outcome data by phone survey (and electronic medical record review) at 12-months postpartum (124 in the early group and 114 in the interval group) (Figure).

**Figure:**
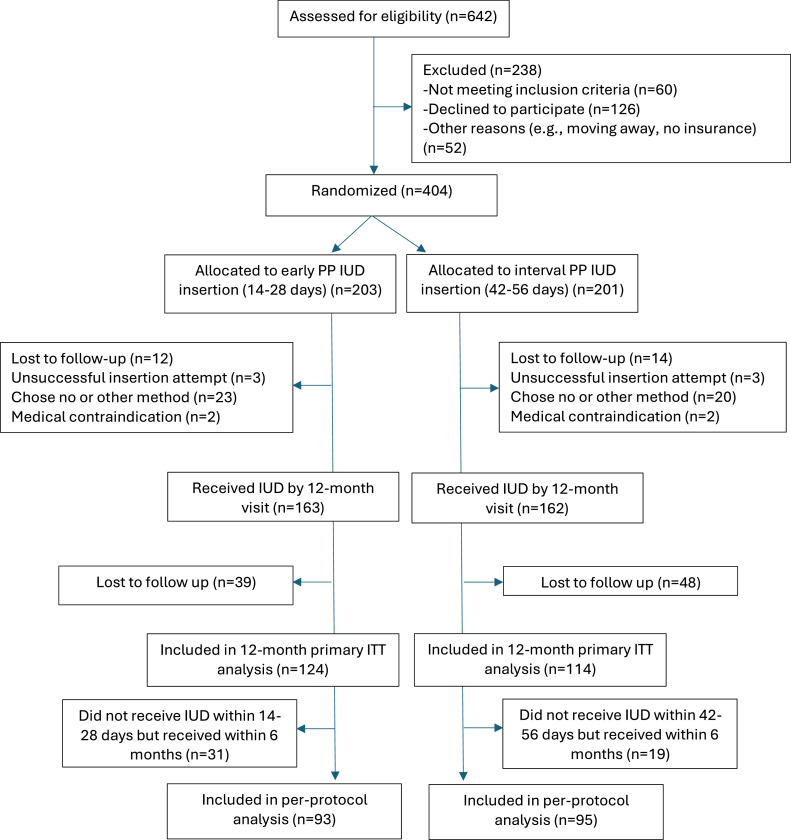
Participant flow diagram in the EPP IUD trial

Averbach et al. Expulsion rates 12 months after early versus interval postpartum intrauterine device placement. Am J Obstet Gynecol 2025.

Baseline characteristics of the 12-month analysis cohort and characteristics of the total 404 study participants were similar between groups (Table 1). The majority identified as white (58.9% early, 58.8% interval); 33.9% in the early group and 40.4% in the interval group identified as Hispanic or Latina. The majority were multiparous (66.1% early, 61.4% interval); 75 % in both groups had a vaginal delivery. Approximately 1 in 3 IUD users chose a copper IUD (37.1 % early, 30.7% interval). Of the remaining hormonal IUD users, the majority chose a 52 mg LNG (levonorgestrel) IUDs (59.7% early, 68.4% interval). Median days to IUD placement was 21 days postpartum in the early group and 45 days postpartum in the interval group.

**Table 1:** Characteristics of participants at baseline/IUD placement (N=404)

		Full cohort N = 404 (%)		Primary ITT cohort^b^ N = 238 (%)	
Characteristics of participants		Early (2-4 weeks) N = 203	Interval (6-8 weeks) N = 201	Early (2-4 weeks) N = 124	Interval (6-8 weeks) N = 114
**Age, years (mean, SD)**		29.8 ±5.2	30.0±5.5	30.9±5.0	30.5±5.4
**Ethnicity**	Hispanic/Latina	82 (40.4)	93 (46.3)	42 (33.9)	46 (40.4)
**race**	Asian, American Indian, Alaska Native, Native Hawaiian, Pacific Islander	18 (8.9)	15 (7.5)	12 (9.7)	10 (8.8)
	Black or African American	26 (12.8)	20 (10.0)	17 (13.7)	11 (9.6)
	Multiracial or other	42 (20.7)	48 (23.9)	21 (16.9)	24 (21.1)
	Refused/declined	3 (1.5)	4 (2.0)	1 (0.8)	2 (1.8)
	White	114 (56.2)	114 (56.7)	73 (58.9)	67 (58.8)
**Education**	Some college or less	111 (54.7)	122 (60.7)	51 (41.1)	58 (50.9)
	College Degree	43 (45.3)	38 (39.3)	30 (24.2)	26 (22.8)
	Graduate Degree	49 (24.1)	41 (20.4)	43 (34.7)	30 (26.3)
**Employment**	full-time	100 (49.3)	92 (45.8)	70 (56.5)	62 (54.4)
	Unemployed, student	81 (39.9)	85 (42.3)	42 (33.9)	37 (32.5)
	part-time	22 (10.8)	24 (11.9)	12 (9.7)	15 (13.2)
**Type of most recent delivery**	Vaginal	155 (76.4)	152 (75.6)	93 (75.0)	86 (75.4)
	Cesarean	48 (23.7)	49 (24.4)	31 (25.0)	28 (24.6)
**Parity**	Primiparous	68 (33.5)	71 (35.3)	42 (33.9)	44 (38.6)
**Type of IUD (among those with an IUD)**		N=163	N=162		
	Copper	56 (34.4)	49 (30.2)	46 (37.1)	35 (30.7)
	52 mg LNG^a^	101 (62.0)	111 (68.5)	74 (59.7)	78 (68.4)
	19.5 mg/13.5 mg LNG^a^	6 (3.7)	2 (1.2)	4 (3.2)	1 (0.9)
**Ultrasound used**		N=163	N=162		
	During placement	31 (19.0)	4 (2.5)	25 (20.2)	1 (0.9)
	After placement only	4 (2.5)	5 (3.1)	2 (1.6)	5 (4.4)
**Lactating at time of insertion**^c^		N=156	N=151	N=120	N=109
	Yes	139 (89.1)	127 (84.1)	107 (89.2)	93 (85.3)
	No	17 (10.9)	24 (15.9)	13 (10.8)	16 (14.7)
**Inserting provider type**^c^		N=162	N=157	N=124	N=111
	Faculty physician	93 (57.4)	75 (47.8)	75 (60.5)	56 (50.5)
	Advanced practice clinician	51 (31.5)	50 (31.8)	33 (26.6)	32 (28.8)
	Resident physician	18 (11.1)	32 (20.4)	16 (12.9)	23 (20.7)

a *LNG*, Levonorgestrel

b *ITT*, Intention to Treat includes all participants with follow-up at the 6-month follow-up visit that had an IUD placed at any time between randomization and 6-month follow up

c Excluding missing values on lactation status and provider type.

Among the modified intention-to-treat cohort, complete expulsion rates at 12 months were 4 in 124 (3.2%; 95% confidence interval [CI], 0.9 to 8.1) and 0 in 114 (0%; 95% CI, 0 to 3.8) in the early and interval groups, respectively, a between-group difference of 3.2 percentage points (95% CI, −0.13 to 8.0), *P*=.054 (Table 2). Partial expulsion rates were 16 (12.9%; 95% CI, 7.6 to 20.1) and 13 (11.4%; 95% CI, 6.2 to 18.7) the early and interval groups, a between-group difference of 1.5 percentage points (95% CI, −7.2 to 10.2), *P*=.75. IUD malposition rates were 6.5% (95% CI, 2.8 to 12.3) and 0% (95% CI, 0.00 to 3.2) in the early and interval groups, a between-group difference of 6.5 percentage points (95% CI, 2.5–12.3), *P*=.01.

**Table 2:** Primary clinical outcomes among early and interval users of IUD at 12-month follow-up visit

	Early (2-4 weeks) (N = 124)	Interval (6-8 weeks) (N = 114)		
Outcome	n (%) [95% CI]	n (%) [95% CI]	Risk difference [95% CI]	*P*-value
**Expulsion**				
Complete expulsion	4 (3.2) [0.9 to 8.1]	0 (0.0) [0.0 to 3.2]	3.2 [−0.1 to 8.0]	.054
Partial expulsion	16 (12.9) [7.6 to 20.1]	13 (11.4) [6.2 to 18.7]	1.5 [−7.2 to 10.2]	.754
Any expulsion	20 (16.1) [10.1 to 23.8]	13 (11.4) [6.2 to 18.7]	4.7 [−4.3 to 13.8]	.305
**Malposition**	8 (6.5) [2.8 to 12.3]	0 (0.0) [0 to 3.2]	6.5 [2.5 to 12.3]	.007
**Perforation**	0 (0.0) [0.0 to 2.9]	1 (0.9) [0.0 to 4.8]	−0.9 [−4.8 to 2.1]	.363

Superiority analysis (Primary intention to treat) (N=238).

We documented three pelvic infections, all in the early group (Table 3); two were diagnosed by the 6-month postpartum visit. One participant reported an asymptomatic chlamydia infection that was treated; one was diagnosed with postpartum endometritis during IUD placement, so the IUD was removed on the same day and the patient was treated with antibiotics. We identified one additional pelvic infection in the early group, 11 months after her IUD was placed. She was diagnosed with PID based on pelvic pain and was treated with antibiotics. The IUD was not removed and she tested negative for sexually transmitted infections. A fourth pelvic infection in the (early/interval) group was documented at 6 months; that participant was lost to follow-up at 12 months (7).

**Table 3:** Secondary clinical outcomes among early and interval users of IUD at 12-month follow-up visit

	Early (2-4 weeks) (N = 124)	Interval (6-8 weeks) (N = 114)		
Outcome	n (%) [95% CI]	n (%) [95% CI]	Risk difference^d^ [95% CI]	*P*-value
**Infection**	3 (2.4) [0.5 to 6.9]	0 (0.0) [0 to 3.2]	2.4 [−0.8 to 6.9]	.108
**IUD removals (including for expulsion/malposition)**	28 (22.6) [15.6 to 31.0]	25 (21.9) [14.7 to 30.6]	0.7 [−10.1 to 11.3]	.921
**Satisfaction with IUD use**				
Satisfied/ very satisfied	107 (86.3) [79.0 to 91.8]	87 (76.3) [67.4 to 83.8]	10.0 [−0.1 to 20.0]	.048
Would recommend IUD to a friend	115 (92.7) [86.7 to 96.6]	97 (85.1) [77.2 to 91.1]	7.7 [−0.4 to 16.0]	.060
Would recommend IUD insertion at the same time to a friend	104 (83.9) [76.2 to 89.9]	99 (86.8) [79.2 to 92.4]	−3.0 [−12.2 to 6.3]	.542
**Strings trimmed (either at string check visit or 6 month)**	18 (14.5) [8.8 to 22.0]	8 (7.0) [3.1 to 13.4]	7.5 [−0.6 to 15.9]	.068

Superiority analysis (Primary intention to treat) (N=238).

Among the total cohort of 404 participants, we diagnosed 16 pregnancies that occurred within 360 days of the index delivery (10 early, 6 interval) (Table 4). Of these, 8 participants in the early group and 5 in the interval group either did not receive an IUD or had the IUD removed prior to the pregnancy. Of the three who became pregnant with an IUD in place, in the early group one hormonal IUD was found to be a complete expulsion and another hormonal IUD was found to be malpositioned. In the interval group one copper IUD was found to be partially expelled. Both of the expulsions and the malpositioned IUD were diagnosed at the time of pregnancy confirmation. The pregnancy that occurred in the setting of the malpositioned IUD was an ectopic pregnancy.

**Table 4:** Pregnancy outcomes among early and interval users of IUD at 12-month follow-up visit

	Early (2-4 weeks) (N = 203)	Interval (6-8 weeks) (N = 201)	Risk Difference [95% CI]	*P*-value
Outcome	n (%)	n (%)		
**Total pregnancies**	**10 (4.9) [2.39 to 8.87]**	**6 (3.0) [1.10 to 6.38]**	**1.94 [-0.02 to 0.06]**	***P*=.337**
Those who received IUD	2	1		
No IUD placement or removed before pregnancy	8	5		

Superiority analysis (Primary intention to treat) (N=404).

We found one perforation between 6 and 12 months among a participant in the interval group. The patient was asymptomatic, had previously been lost to follow up at 6 months, and was not aware that the IUD had perforated until it was incidentally diagnosed on abdominal imaging for another reason. She underwent an uncomplicated laparoscopic IUD removal.

Most participants were satisfied or very satisfied with their IUD; however, satisfaction was higher among those in the early compared to interval groups (86.3% early, 95% CI 79.0 to 91.8; 76.3% interval, 95% CI 67.4 to 83.8; *P*=.048).

Among IUD users, 53 participants removed their IUD throughout the duration of the study: 28 in the early group (22.6%, 95% CI 15.6 to 31.01) and 25 in the interval group (21.9%, 14.7 to 30.6); 11 chose to remove their IUD because of pain (2 in the early group and 9 in the interval group) and 6 for bleeding (1 in the early group and 5 in the interval group). One participant (early group) removed the IUD because she desired another pregnancy and 8 participants chose removal for another reason (participants were able to choose multiple reasons). The remaining removals were due to partial expulsion (27), malposition (8) and perforation (1) as described above.

Twenty-six participants reported having their IUD strings trimmed after placement, 18 in the early group (14.5%; 95% CI 8.8 to 22.0) compared to 8 participants in the interval group (7.0%; 95% CI 3.1 to 13.4), *P*=.07).

Among the full sample of 404, IUD utilization at 12 months postpartum was higher among those with IUDs placed in the early group compared to the interval group; however, the difference was not statistically significant (113, 55.7%; 95% CI 48.5 to 62.6) and (95, 47.3%; 95% CI 40.2 to 54.4) *P*=.10.

There were no meaningful differences in the per-protocol analyses compared to the intent to treat analysis (Supplemental Table 1 and 2).

## Comment

### Principal findings

We found that the risk of complete IUD expulsion was low at 12 months postpartum when IUDs were placed in the early and interval postpartum periods. There may be a small absolute difference in the rate of complete IUD expulsion after early compared to interval placement (3.2% vs 0%). While this difference did not meet statistical significance (*P*=.054) we may have been underpowered to detect small differences between groups. Our power calculation was not designed for this secondary outcome analysis at 12 months postpartum. In addition, loss to follow-up, although common among postpartum people, was even larger than expected. The upper boundary of the 95% CI around the risk difference suggests that early placement confers only modestly higher risk of complete expulsion than interval placement, at most 8%. The possibility of a small increase in the risk of expulsion should be weighed against the risk of pregnancy before interval placement and contextualized by patient goals and preferences.

We found that IUD utilization was at least as high after early compared to interval placement (56% vs 47%, *P*=.10) despite the small difference in expulsion. Participants were also more satisfied with early placement compared to interval placement (86% vs 76%, *P*=.048). Therefore, the differential rates of expulsion did not have a meaningful impact on utilization or satisfaction.

Congruent with our findings at 6-month follow-up, we found more malpositioned IUDs in the early group compared to the interval group (6.5% vs 0%, *P*=.01). However, the clinical significance of malpositioned IUDs is also unclear, especially in the absence of symptoms.^11^, ^12^, ^13^, ^14^

### Results in the context of what is known

IUDs are typically placed 6 weeks after birth; however, immediate postpartum (IPP) IUD placement, within 10 minutes of delivery, has also been shown to be safe and acceptable to patients and providers.^15^ Despite the benefits of IPP IUD placement, significant barriers prevent widespread implementation, most importantly a lack of reimbursement mechanisms for inpatient devices and training for immediate postpartum placement.^16^ Although efforts are underway to increase access to IPP IUDs, they are developing slowly. Additional strategies, such as offering IUDs in the EPP period, are urgently needed to allow for flexibility and multiple options for provision of highly effective contraception in the postpartum period.

Both immediate and interval IUD placement carry unique risks. IPP IUD placement is associated with an increased risk of expulsion, with rates as high as 10% to 26% compared to 2% to 5% for IUDs placed at 6 weeks postpartum.^6^ Studies have shown that approximately 80% of uterine involution occurs by 14 days postpartum^17^^,^^18^ which could be protective against IUD expulsion after 14 days compared to immediate placement, and which may explain why we found complete expulsion rates in the EPP placement group that were much lower than historical rates at the time of IPP placement.^6^ A large cohort study also found that expulsion rates were highest among people with IUDs inserted in the IPP period and lower when IUDs were placed between 4 days and 6 weeks postpartum.^19^

Interval IUD placements are associated with a small absolute increase in the risk of perforation compared to IUD placements among nonpostpartum people.^20^ Although we were not powered to detect meaningful differences in rare outcomes, specifically perforation, it is encouraging that there were no perforations identified in in the early group and only one in the interval group. This finding suggests that the rate of perforations is low when IUDs are placed in the early and interval postpartum periods, and there was no signal for increased risk for perforation with early IUD placement.

### Clinical implications

Our data demonstrate that IUD placement between 2 and 4 weeks postpartum is associated with low rates of IUD expulsion in the entire first year after delivery. The rates of expulsion of IUDs placed in the EPP period are also significantly lower than historical expulsion rates for immediate postpartum placement. There were no perforations in the early group and only one in the interval group.

### Research implications

Further data are needed to better understand if the risk of IUD expulsion differs by week in the early postpartum period, especially whether the risk of expulsion is greater in the first week postpartum before the majority of uterine involution has occurred. More research is needed to understand the role of ultrasound guidance in mitigating risk of IUD expulsion when IUDs are placed in the early postpartum period.

### Strengths and limitations

Our study had several strengths. It is a randomized trial designed to assess IUD expulsion in the early postpartum period. The study population included participants from urban, rural, suburban, and military sites. IUDs were placed by a variety of clinicians including advanced practice clinicians (nurse practitioners and certified nurse midwives) and resident physicians. Therefore, our data is widely generalizable to many settings.

Our results should be considered in the context of several limitations. The rate of complete IUD expulsion was lower than anticipated in both groups. In addition, a greater proportion of people than anticipated chose not to have an IUD inserted after randomization, and we had greater than expected loss to follow-up (27% at 1 year). Therefore, there may be small differences between groups that we were underpowered to detect. Follow-up at 6-months postpartum was in person, however, follow-up at 12-months postpartum was by telephone. It is possible that a participant could have had an unrecognized expulsion that we are unable to ascertain by telephone. However, it should be noted that all IUD expulsions that occurred within the first 6 months were recognized by participants. It is unlikely there were a large number of unrecognized expulsions at 12-months.

A greater proportion of IUDs were placed under ultrasound in the early group compared to the interval group (20% vs, 1%, *P*<.001). However, in the primary analysis, ultrasound use was associated with greater risk of expulsion in logistic regression; therefore, it is unlikely that use of ultrasound use resulted in lower risk of IUD expulsion in the early group than it would have been without ultrasound.^7^ Finally, although our study was pragmatic in design, our data were generated in the context of a randomized trial conducted at academic medical centers and may not be generalizable to community settings.

## Conclusion

Understanding the risk of expulsion when IUDs are placed 2 to 3 weeks or 4 to 6 weeks postpartum will enable patients and clinicians to use shared-decision making to make informed choices about when to initiate postpartum IUDs based on patient’s goals and preferences. Offering IUDs at many times during the postpartum period can help postpartum people achieve their reproductive goals, prevent unintended pregnancy, and support healthy birth spacing.

## CRediT authorship contribution statement

**Sarah Averbach:** Conceptualization, Data curation, Formal analysis, Funding acquisition, Investigation, Methodology, Project administration, Supervision, Writing – original draft, Writing – review & editing. **Florin Vaida:** Data curation, Formal analysis, Supervision, Writing – review & editing. **Erica Hinz:** Data curation, Project administration, Supervision, Writing – review & editing. **Gennifer Kully:** Investigation, Project administration, Writing – review & editing. **Arnab K. Dey:** Formal analysis, Methodology, Software, Validation, Writing – review & editing. **Monica A. Lutgendorf:** Investigation, Resources, Writing – review & editing. **Sadia Haider:** Investigation, Methodology, Supervision, Writing – review & editing. **Lisa G. Hofler:** Conceptualization, Investigation, Methodology, Project administration, Resources, Supervision, Writing – review & editing.

## Appendix Supplementary materials

Image, application 1

## Footnotes

Supplementary material associated with this article can be found, in the online version at doi:10.1016/j.xagr.2025.100547.
