# Supplementary material for: Expulsion rates 12 months after early versus interval postpartum intrauterine device placement: a randomized trial
Source: AJOG Glob Rep. 2025 Jul 20;5(3):100547. doi: 10.1016/j.xagr.2025.100547 (PMC12391790; doi:10.1016/j.xagr.2025.100547)
Supplement: Supplementary file 1 [file mmc1.docx]

**Supplemental table 1: Primary clinical outcomes among early and interval users of IUD at 6-month follow-up visit; Superiority analysis (Per Protocol Analysis) (N=188)**

|  | **Early**  **(2-4 weeks)**  **(N = 93)** | **Interval**  **(6-8 weeks)**  **(N = 95)** |  |  |
| --- | --- | --- | --- | --- |
| **Outcome** | **n (%)**  **[95% CI]** | **n (%)**  **[95% CI]** | **Risk Difference**  **[95% CI]** | **p-value** |
| **Expulsion** |  |  |  |  |
| Complete Expulsion | 3 (3.2)  [0.7 to 9.1] | 0 (0.0)  [0.0 to 3.8] | 3.2  [-0.8 to 9.1] | 0.07 |
| Partial Expulsion | 15 (16.1)  [9.3 to 25.2] | 10 (10.5)  [5.2 to 18.5] | 5.6  [-4.4 to 15.8] | 0.27 |
| Any expulsion | 18 (19.4)  [11.9 to 28.9] | 10 (10.5)  [5.2 to 18.5] | 8.8  [-1.6 to 19.4] | 0.09 |
| **Malposition** | 4 (4.3)  [1.2 to 10.6] | 0 (0.0)  [0 to 3.8] | 4.3  [0.2 to 10.6] | 0.04 |
| **Perforation** | 0 (0.0)  [0.0 to 3.9] | 1 (1.1)  [0.0 to 5.7] | -1.1  [-5.8 to 2.9] | 0.51 |

|  | **Early**  **(2-4 weeks)**  **(N = 93)** | **Interval**  **(6-8 weeks)**  **(N = 95)** |  |  |
| --- | --- | --- | --- | --- |
| **Outcome** | **n (%)**  **[95% CI]** | **n (%)**  **[95% CI]** | **Risk Difference**  **[95% CI]** | **p-value** |
| **Infection** | 2 (2.2)  [0.3 to 7.6] | 0 (0.0)  [0 to 3.8] | 2.2  [-1.8 to 7.6] | 0.16 |
| **IUD Removals** | 21 (22.6)  [14.6 to 32.4] | 21 (22.1)  [14.2 to 31.8] | 0.5  [-11.6 to 12.6] | 0.99 |
| **Satisfaction with IUD use** |  |  |  |  |
| Satisfied/ Very satisfied | 82 (88.2)  [79.8 to 93.9] | 70 (73.7)  [63.6 to 82.2] | 14.5  [3.2 to 25.9] | 0.01 |
| Would recommend IUD to a friend | 90 (96.8)  [90.9 to 99.3] | 80 (84.2)  [75.3 to 90.9] | 12.6  [4.3 to 21.1] | 0.00 |
| Would recommend IUD insertion at the same time to a friend | 79 (84.9)  [76.0 to 91.5] | 83 (87.4)  [79.0 to 93.3] | -2.4  [-12.8 to 7.8] | 0.65 |
| **Strings Trimmed (either at string check visit or 6 month)** | 17 (18.3)  [11.0 to 27.6] | 7 (7.4)  [3.0 to 14.6] | 10.9  [1.3 to 21.1] | 0.03 |

**Supplemental Table 2: Secondary clinical outcomes among early and interval users of IUD**

**at 6-month follow-up visit; Superiority analysis (Per-Protocol) (N=188)**
